# Kinetic analysis of antagonist-occupied adenosine-A_3_ receptors within membrane microdomains of individual cells provides evidence of receptor dimerization and allosterism

**DOI:** 10.1096/fj.13-247270

**Published:** 2014-10

**Authors:** Ross Corriden, Laura E. Kilpatrick, Barrie Kellam, Stephen J. Briddon, Stephen J. Hill

**Affiliations:** * Institute of Cell Signalling, School of Life Sciences, Medical School, and; † School of Pharmacy, Centre for Biomolecular Sciences, University of Nottingham, Nottingham, UK

**Keywords:** GPCR, dissociation, fluorescence correlation spectroscopy, fluorescent ligand

## Abstract

In our previous work, using a fluorescent adenosine-A_3_ receptor (A_3_AR) agonist and fluorescence correlation spectroscopy (FCS), we demonstrated high-affinity labeling of the active receptor (R*) conformation. In the current study, we used a fluorescent A_3_AR antagonist (CA200645) to study the binding characteristics of antagonist-occupied inactive receptor (R) conformations in membrane microdomains of individual cells. FCS analysis of CA200645-occupied A_3_ARs revealed 2 species, τ_D2_ and τ_D3_, that diffused at 2.29 ± 0.35 and 0.09 ± 0.03 μm^2^/s, respectively. FCS analysis of a green fluorescent protein (GFP)-tagged A_3_AR exhibited a single diffusing species (0.105 μm^2^/s). The binding of CA200645 to τ_D3_ was antagonized by nanomolar concentrations of the A_3_ antagonist MRS 1220, but not by the agonist NECA (up to 300 nM), consistent with labeling of R. CA200645 normally dissociated slowly from the A_3_AR, but inclusion of xanthine amine congener (XAC) or VUF 5455 during washout markedly accelerated the reduction in the number of particles exhibiting τ_D3_ characteristics. It is notable that this effect was accompanied by a significant increase in the number of particles with τ_D2_ diffusion. These data show that FCS analysis of ligand-occupied receptors provides a unique means of monitoring ligand A_3_AR residence times that are significantly reduced as a consequence of allosteric interaction across the dimer interface.—Corriden, R., Kilpatrick, L. E., Kellam, B., Briddon, S. J., Hill, S. J. Kinetic analysis of antagonist-occupied adenosine-A_3_ receptors within membrane microdomains of individual cells provides evidence for receptor dimerization and allosterism.

The adenosine-A_3_ receptor (A_3_AR) is a G-protein-coupled receptor (GPCR) and the most recently identified subtype of the adenosine receptor family (1). It is widely distributed throughout the body and has been proposed as a target for therapeutic intervention in several diseases, including inflammation, glaucoma, and cancer (2–7). More recently, this receptor has been shown to be expressed in a highly localized manner in human neutrophils and may play an important role in the control of infection (8). In these cells, the A_3_AR is concentrated at the leading edge during chemotaxis, where it promotes cell migration (9, 10) and the formation of filopodia-like projections that can scavenge pathogens (8).

Discrete organization of GPCRs, such as the A_3_AR, in membrane compartments and microdomains is becoming increasingly evident (11–14). This spatial segregation provides a mechanism by which intracellular signaling can be orchestrated within a cell in a manner dependent on the signaling proteins resident within the specific membrane domains containing the receptor. This mechanism may also lead to a signaling bias, whereby specific agonists can direct signaling to either classic G-protein-coupled signaling pathways or to G protein-independent pathways (*e.g.*, β-arrestin) *via* the same receptor (15–18) and may be a consequence of ligands interacting in different ways with the key elements of the protein responsible for receptor activation (19, 20). However, biased signaling may also be an extension of the concept of allosterism (2, 17, 21, 22), whereby a signaling protein (*i.e.*, G protein or β-arrestin) can bind to a GPCR at a site distinct from the orthosteric ligand-binding site for the natural agonist (in this case, adenosine) and produce a conformational change. This allosteric effect may also alter the efficacy or affinity of a ligand's binding to the orthosteric site in a probe-dependent manner (2, 17, 21, 22).

Fluorescent ligands for several GPCRs (23–27) have been successfully used to produce evidence of allosterism at the single-cell level (28–30). These studies have shown that allosteric ligands and negative cooperativity across A_3_AR homodimers can produce marked changes in the dissociation kinetics of fluorescent ligands from the orthosteric site of the receptor (28–30). Fluorescence correlation spectroscopy (FCS) is a quantitative biophysical technique that can be used to quantify both the diffusional characteristics and number of fluorescent ligand-occupied receptor complexes within a highly localized membrane microdomain (∼0.2 μm^2^; ref. 29). Fluorescent ligands, in combination with FCS, have also been used to study the properties and behavior of adenosine receptors in discrete membrane microdomains of living cells (14, 23, 32–34).

Previous FCS work with a fluorescent A_3_AR agonist revealed 2 populations of agonist-occupied A_3_AR complexes with apparently different diffusion coefficients and molecular sizes (diffusion coefficients of 2.7 and 0.12 μm^2^/s; ref. 14). Competition studies with an antagonist (MRS 1220) and agonist (NECA) indicated that the slower diffusing component had high (low nanomolar) affinity for both agonists and antagonists. This finding suggests that the use of very low concentrations of fluorescent agonist in that study allowed selective labeling of an active receptor (R*) conformation of the A_3_AR (14). Furthermore, the lack of effect of pertussis toxin on this high-affinity agonist binding suggested that the agonist-occupied receptor detected was not coupled to G_i_ proteins (14). Unfortunately, the amount of the faster component labeled by the fluorescent agonist was too low for detailed analysis, although it was speculated that it might represent the inactive receptor (R) conformation, which normally has low affinity for agonists (14).

To further clarify this question, in the current study we used FCS to investigate the diffusional characteristics of antagonist–A_3_AR complexes in membrane domains by using the fluorescent antagonist CA200645 (27), which should have a high affinity for R. We used this approach to investigate the nature of the faster diffusing species that was detected in a prior study with a fluorescent agonist (14). We also provide evidence that simultaneous monitoring of the 2 diffusing species detected with a fluorescent antagonist can yield insights into receptor allosterism, ligand residence time, and dimerization in small membrane microdomains.

## MATERIALS AND METHODS

### Materials

MRS 1220 was obtained from Tocris Cookson (Avonmounth, UK). CA200645 and ABEA-X-BY630 were obtained from CellAura Technologies (Nottingham, UK). VUF 5455 was synthesized by B. Kellam (Centre for Biomolecular Sciences, School of Pharmacy, University of Nottingham, Nottingham, UK). Fetal calf serum was obtained from PAA Laboratories (Yeovil, UK). All other reagents, including xanthine amine congener (XAC), were obtained from Sigma-Aldrich Inc. (Poole, UK).

### Cell culture

CHO-K1 cells stably expressing either the human A_3_AR (CHO-A_3_ cells; ref. 14) or an A_3_AR–green fluorescent protein (GFP) fusion protein (CHO-A_3_GFP cells) were used. The CHO-A_3_ GFP cell line was generated by transfecting CHO-K1 cells with a pcDNA3.1 plasmid containing cDNA encoding the full-length human A_3_AR fused in frame with GFP by using Lipofectamine (Life Technologies, Paisley, UK) according to the manufacturer's instructions. The transfected cells were subjected to selective pressure for 2–3 wk by the addition of 1 mg/ml G418 to the normal growth medium to generate a stable, mixed-population cell line. For confocal and FCS analysis, the cells were grown in phenol red–free Dulbecco's modified Eagle's medium/Ham's F-12 (DMEM-F12) containing 10% fetal calf serum and 2 mM glutamine and incubated under the same conditions; 48 h before experimentation, the cells were seeded in Nunc Labtek 8-well plates (Fisher Scientific, Loughborough, UK). Before the analysis, the cells were washed twice with HEPES-buffered saline solution (HBSS; ref. 23), which also served as the incubation medium in the experiments.

### Confocal imaging

Live-cell imaging was performed with CHO-A_3_ or CHO-A_3_-GFP cells grown in Nunc Labtek 8-well plates and maintained as described above. For the ligand-binding experiments, the cells were incubated with the required concentration of fluorescent ligand for 10 min at 37°C before imaging. Binding specificity was assessed by preincubating cells with the nonfluorescent A_3_AR antagonist MRS 1220 (100 nM) for 30 min at 37°C before the addition of CA200645. Images were captured with a Zeiss LSM710META confocal microscope (Zeiss, GmbH, Jena, Germany) fitted with a Plan-Apochromat ×63, 1.40 NA, DIC, oil-immersion objective (Zeiss). A 488 nm argon laser was used to excite the GFP, and a 633 nm HeNe laser was used to excite the BODIPY 630/650-labeled CA200645. A variable spectral detection system was used to capture emission at 480–530 and 645–680 nm for GFP and CA200645, respectively. Images within each set of experiments were collected by using identical settings for pinhole diameter, laser power, detector gain, and offset.

### FCS

On the day of experimentation, CHO-A_3_ cells were washed twice in HBSS before further incubation in the presence or absence of MRS 1220 (0.3–300 nM; 20 min at 37°C) or the nonselective adenosine receptor agonist NECA (0.3–300 nM; 10 min at 24°C). Cells were allowed to equilibrate to 22°C and were then exposed to 1, 2.5, 5, or 10 nM CA200645 for 10 min. FCS measurements were then taken on the upper membrane of individual cells with a Confocor2 fluorescence correlation spectrometer (Zeiss) with a c-Apochromat ×40, 1.2 NA, water-immersion objective, as described elsewhere (23). Briefly, the detection volume was localized in the *x–y* plane over the cell nucleus, with a live transmitted light image, and subsequently in the *z* plane, with an intensity scan. For FCS measurements with fluorescently labeled ligands, fluorescence fluctuations were recorded for two 30 s intervals at a laser power of 0.3 kW/cm^2^ following a 10 s prebleaching step at a laser power of 0.2 kW/cm^2^. For measurements using GFP-tagged receptors, fluorescence fluctuations were recorded for two 30 s intervals at a laser power of 0.15 kW/cm^2^ following a 10 s prebleaching at 0.05 kW/cm^2^. FCS measurements of CA200645 in solution were also made, to determine the diffusion coefficient of free ligand (9 times for 10 s each, 0.15 kW/cm^2^). Fluorescence fluctuations were evaluated with standard autocorrelation analysis within the Zeiss AIM 4.2 software.

The autocorrelation function *G*(τ) for fluctuations around a mean intensity *I* is described as follows, with the angle brackets representing an ensemble average:

$$G\left(\tau \right)=1+\frac{〈\delta I(t)\cdot \delta I(t+\tau )〉}{〈I^{2}〉}$$

Here, the intensity fluctuation δ*I*(*t*) around the average intensity *I*, at time *t*, is correlated with the fluctuation at a given time later, δ*I*(*t*+τ). The algebraic form of this equation, relating to 3-dimensional (3D) diffusion of several fluorescent species through a gaussian volume, is

$$G\left(\tau \right)=1+\frac{1}{N}\overset{m}{\underset{i=1}{\sum }}f_{i}{\left(1+\frac{\tau }{\tau_{\text{D}i}}\right)}^{-1}{\left(1+\frac{\tau }{S^{2}\cdot \tau_{\text{D}i}}\right)}^{-1/2}$$

where *f_i_* is the fraction of species *i*, from a total number of species *m*, with a mean dwell time in the volume of τ_D*i*_; *N* is the number of fluorescent particles in the volume; and *S* is a structure parameter, representing the ratio of the radial and vertical axes ω_1_ and ω_2_, respectively, of the confocal volume. For a species diffusing in 2 dimensions, such as a membrane receptor, *S* → ∞, an algebraic form of the autocorrelation equation simplifies to

$$G\left(\tau \right)=1+\frac{1}{N}\sum_{i=1}^{m}f_{i}{\left(1+\frac{\tau }{\tau_{\text{D}i}}\right)}^{-1}$$

Nonlinear curve fitting of autocorrelation curves, using these equations or combinations thereof to account for multiple 2D and 3D diffusing components, yields values for *N*, *f_i_*, and τ_D*i*_ for each component. The confocal measurement volume *V*_C_ was then estimated as follows:

$$V_{\text{C}}=\pi^{3/2}\cdot \omega_{1}\cdot \omega_{2}$$

allowing the concentrations of each fluorescent species to be calculated.

For FCS binding experiments, the number of particles *N* and average dwell times τ_*D*_ were calculated from autocorrelation curves that were generated at the upper membrane of the CHO cells with fluorescent ligands. Autocorrelation curves were fitted to a model containing one 3D component (τ_D1_, representing free fluorescent ligand) and two 2D diffusion components (τ_D2_ and τ_D3_, representing bound ligand), in addition to a preexponential term to account for the triplet state of the fluorophore. Binding was quantified by using the value of *N* obtained from the fitted autocorrelation curve and the appropriate contribution of the identified component (τ_D2_ or τ_D3_). Total binding represents the sum of the τ_D2_ and τ_D3_ components. The value for τ_D1_ was fixed during fitting to that determined for free ligand in HBSS. For measurements of the A_3_AR-GFP construct, results were fitted to a model including two 2D diffusion components (32).

Calibration of the system allowed quantification of diffusion coefficients and the number of particles, as described in Results. The radius of the detection volume at the beam waist (ω_1_) was calculated by determining the mean dwell times of aqueous solutions of rhodamine 6G (Rh6G) for the 488 nm laser and of Cy5 NHS ester for the 633 nm laser, as follows:

$$\omega_{1}={\left(4\cdot \tau_{\text{D}}\cdot D\right)}^{1/2}$$

where *D* is 3.16 × 10^−6^ cm^2^/s for Cy5 and 2.80 × 10^−6^ cm^2^/s for Rh6G. Average ω_1_ values were 0.16 and 0.27 μm for the 488 and 633 nm beam paths, respectively. These values were subsequently used to calculate beam area at the waist (*A* = π · ω_1_^2^) and the particle density (*N*/μm^2^) for each fluorescent component. To account for differences in the size of confocal volumes generated using the 488 and 633 nm lasers, average τ_D_ values were converted to diffusion coefficient *D* (μm^2^/s) values, with the equation *D* = ω_1_^2^/4 · τ_*D*_. The *n* values quoted for the FCS experiments represent the number of cells that were measured in ≥3 independent experiments.

### FCS determination of allosteric interactions

CHO-A_3_ cells were prepared for FCS as described above. After a 10 min preincubation at 22°C with HBSS containing 5 nM CA200645, the medium was removed from the well; after 1 wash with ligand-free HBSS, the medium was replaced with HBSS containing either an allosteric (VUF 5455) or orthosteric [xanthine amine congener (XAC)] ligand at various concentrations. Single, 40 s FCS measurements were taken at 2, 4, and 6 min after medium replacement, with *z* scans performed between reads to ensure proper placement of the confocal volume. The amount of bound fluorescent antagonist was determined at each time point by using the analysis methods described above.

### Statistics

Statistical significance was determined by either Student's unpaired *t* test or ANOVA with the *post hoc* Newman-Keuls or Dunnett analysis. All data are presented as means ± se. Unless otherwise noted, values of *n* refer to the number of separate experiments performed.

## RESULTS

### Validation of CA200645 as a fluorescent A_3_AR antagonist

**Figure 1.**
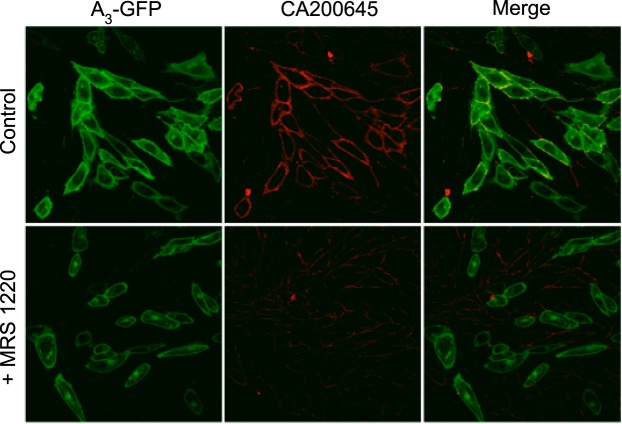
Live-cell confocal imaging of CA200645 binding. CHO-K1 cells stably expressing an A_3_AR-GFP construct (green channel) were incubated with the fluorescent A_3_AR antagonist CA200645 (25 nM; red channel) for 10 min at 22°C (top panels). Cells that were preincubated with the unlabeled A_3_AR antagonist MRS 1220 for 30 min at 37°C before addition of CA200645 exhibited reduced membrane binding of the fluorescent ligand on A_3_AR-GFP-expressing cells (bottom panels). Images are from an individual experiment representative of 3 performed.

CA200645 has been reported to be a high-affinity fluorescent antagonist for human A_3_AR and has been used to label the A_3_AR in live cells (27). **Figure 1** shows the binding of this ligand (25 nM; 10 min at 22°C) to CHO-K1 cells stably transfected with an A_3_AR-GFP construct. Single equatorial confocal images revealed membrane clearly labeled with the red ligand that was colocalized with the GFP fluorescence of the receptor (Fig. 1). The observed membrane binding was substantially reduced by preincubation with MRS 1220 (100 nM, 30 min at 22°C; Fig. 1), indicating that most of the membrane-localized ligand could be attributed to specific binding to A_3_AR-GFP. Notably, the data obtained at temperatures normally used for FCS (22°C) were similar to those previously reported at 37°C (27).

### FCS analysis of GFP-tagged human A_3_ARs

**Figure 2.**
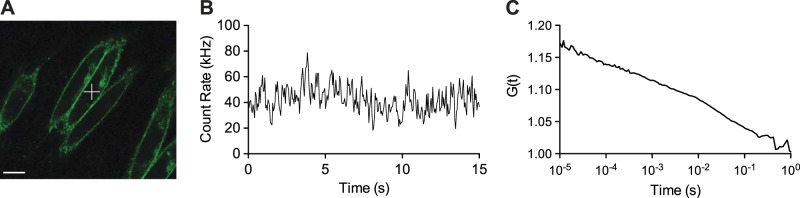
FCS analysis of A_3_AR-GFP-transfected CHO-K1 cells. *A*) Live-cell fluorescence images were used to localize the confocal volume in the *x–y* plane above the nuclei of A_3_AR-GFP-expressing CHO-K1 cells (indicated by white cross). Scale bar = 5 μm. *B*, *C*) The confocal volume was positioned directly on the cell membrane with a *z* scan, and fluctuations in fluorescence intensity were measured (*B*) and subjected to autocorrelation analysis (*C*). Fitting of the autocorrelation curve was performed with a model incorporating two 2D components. This analysis revealed 2 fluorescent species with dwell times of 147 ± 22 μs and 80.1 ± 6.4 ms. As described previously (23), the first represents photophysical effects; the second represents the average dwell time of GFP-tagged A_3_ARs in the confocal volume. Results in *A* and *B* are from 1 cell representative of 64 individual cells measured in 3 independent experiments used to derive the average dwell time (expressed as mean±se).

The biophysical properties of GFP-tagged A_3_ARs (in stably transfected CHO-K1 cells) were investigated with FCS by positioning the confocal volume on the cell membrane directly above the nucleus (**Fig. 2*A***). Autocorrelation analysis of the fluorescence fluctuations (Fig. 2*B*) revealed 2 fluorescent species with dwell times of 147 ± 22 μs and 80.1 ± 6.4 ms (3 individual experiments, *n*=64 cells total). As described previously (23), the first dwell time represents fluctuations generated by intramolecular photophysical effects, such as blinking of the GFP fluorophore, while the second represents the average dwell time of GFP-tagged A_3_ARs in the confocal volume, yielding a diffusion coefficient of 0.105 ± 0.006 μm^2^/s (Fig. 2*C*).

### FCS analysis of CA200645 binding

**Figure 3.**
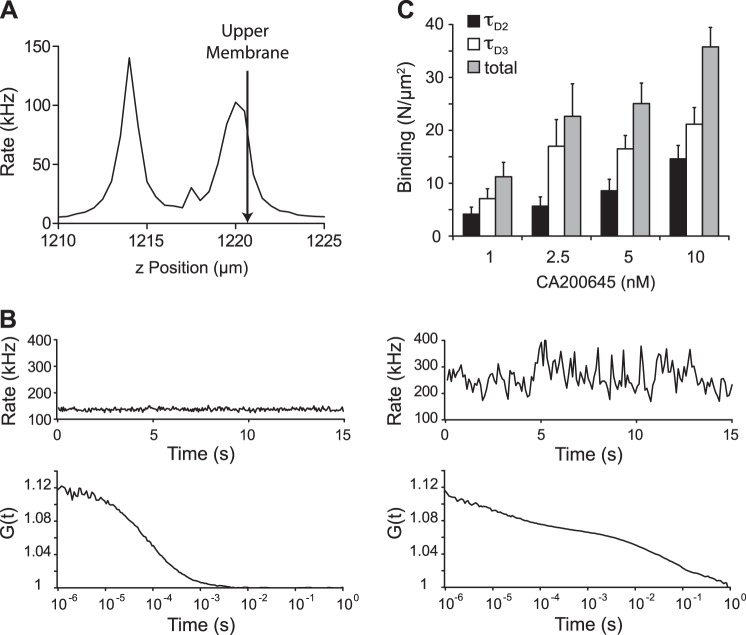
FCS analysis of CA200645 binding to the upper membrane of CHO-A_3_ cells. *A*) To analyze binding of the fluorescent antagonist CA200645 to CHO-A_3_ cells, the confocal volume was localized in the *x–y* plane with a live transmitted light image and subsequently positioned 0.5 μm above the peak intensity on the upper membrane by using a fluorescence intensity *z* scan. *B*) Fluorescence fluctuations (top panels) and their associated autocorrelation curves from measurements of 100 nM CA200645 in HBSS (bottom left panel) and on the upper membrane of a CHO-A_3_ cell incubated with 5 nM CA200645 for 10 min at 22°C (bottom right panel). Autocorrelation analysis of the curve for CA200645 in solution was best fit with a single 3D component curve giving a dwell time, τ_D1_, of 71 μs. Similar analysis of the autocorrelation curve from CHO-A_3_ cell membranes revealed 2 further slow-diffusing components (τ_D2_ and τ_D3_) of 4.8 and 48.6 ms. *C*) CHO-A_3_ cells were incubated with the indicated concentrations of CA200645 (10 min at 22°C), and FCS measurements were taken on the upper membrane as in panel *A*. Autocorrelation analysis was performed as in panel *B*, to quantify the number of CA200645 particles exhibiting characteristics of τ_D2_ (solid bars) and τ_D3_ (open bars), and total binding (τ_D2_+τ_D3_; shaded bars) was determined as described in Materials and Methods. Results are shown as means ± se of 28 cells measured over 3 separate experiments. Average dwell times of the τ_D2_ and τ_D3_ components were 8.7 ± 1.3 and 265.8 ± 41.7 ms, respectively.

FCS was then used to quantify the binding of CA200645 to the A_3_AR and the diffusional characteristics of ligand–receptor complexes in nonspecified membrane microdomains of transfected CHO-K1 cells. Autocorrelation analysis of FCS data generated by positioning the confocal volume in an aqueous solution of CA200645 (100 nM) generated monophasic autocorrelation curves (**Fig. 3*B***, bottom left panel) consisting of a single component (τ_D1_) with an average diffusion coefficient of 257.7 ± 8.9 μm^2^/s (*n*=6). To determine the diffusional characteristics of CA200645/A_3_AR complexes in live cells, we performed an intensity scan to enable positioning of the confocal volume at the upper membrane/extracellular boundary (Fig. 3*A*), which enabled simultaneous detection of free antagonist and antagonist–receptor complexes. The autocorrelation curves that resulted from FCS measurements at the upper membrane after incubation of CHO-A_3_ cells with 10 nM CA200645 (10 min at 22°C) yielded 2 additional, more slowly diffusing, species (Fig. 3*B*, bottom right panel). These 2 slowly diffusing species, τ_D2_ and τ_D3_, exhibited average diffusion coefficients of 2.29 ± 0.35 and 0.09 ± 0.03 μm^2^/s, respectively (*n*=100 cells, 3 separate experiments). Incubation with increasing concentrations of CA200645 elevated the number of both species detected on ∼0.2 μm^2^ of membrane (Fig. 3*C*). It was notable that the number of particles with τ_D3_ diffusional characteristics appeared to saturate over the concentration range used, whereas those for τ_D2_ continued to increase.

### Competitive binding analysis

**Figure 4.**
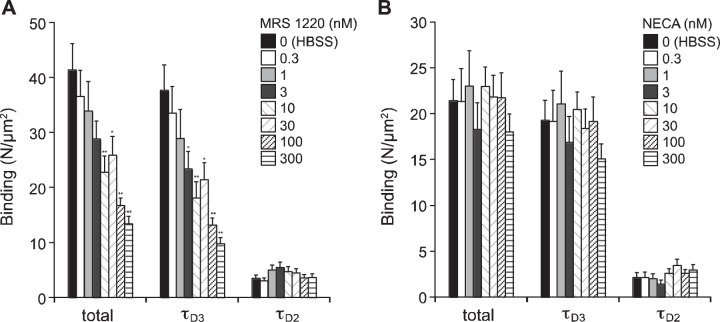
Effect of MRS 1220 and NECA on the formation of antagonist–A_3_AR complexes in the membranes of CHO-A_3_ cells. *A*) CHO-A_3_ cells were incubated with the indicated concentrations of MRS 1220 (20 min at 37°C) before the addition of 5 nM CA200645 (10 min at 22°C). FCS measurements were taken on the cell membrane, and τ_D2_, τ_D3_, and total bound CA200645 (τ_D2_+τ_D3_) were quantified as described in Materials and Methods. *B*) Similar measurements were made using CHO-A_3_ cells incubated with the indicated concentrations of NECA (10 min at 22°C) before the addition of 5 nM CA200645 (10 min at 22°C). Results represent means ± se of 30–32 individual cells taken over 4 independent experiments; analyzed by 1-way ANOVA and the *post hoc* Newman-Keuls test. *P < 0.05, **P < 0.001 *vs.* HBSS-only control.

To further probe the pharmacological properties of CA200645/A_3_AR complexes, we performed competitive binding experiments with CA200645 (5 nM) and unlabeled agonists and antagonists. Quantification of the observed number of particles on the membranes of cells that were preincubated with increasing concentrations of the unlabeled, A_3_AR-selective antagonist MRS 1220 (**Fig. 4*A***) revealed a reduction in the number of slow-diffusing complexes (τ_D3_), whereas no significant difference in the number of faster diffusing (τ_D2_) complexes was observed. The pIC_50_ obtained for MRS 1220 from this analysis was ∼8.5 (Fig. 4*A*), giving an estimate for p*K*i of 9.0 (following a Cheng-Prussoff correction using a p*K*b for CA200645 of 8.5 ref. 27), which is close to the value of 9.3 reported for this A_3_AR antagonist (14). However, in contrast with our findings using the fluorescent A_3_AR agonist ABEA-X-BY630 under the same conditions (14), preincubation with the nonselective adenosine receptor agonist NECA did not result in a significant reduction in the number of slow (τ_D3_)- or fast (τ_D2_)-diffusing complexes detected (Fig. 4*B*). These data suggest that using a fluorescent antagonist ligand under these conditions labels a species with much lower affinity for agonists than when a fluorescent agonist ligand is used.

### Quantification of allosteric interactions by FCS

**Figure 5.**
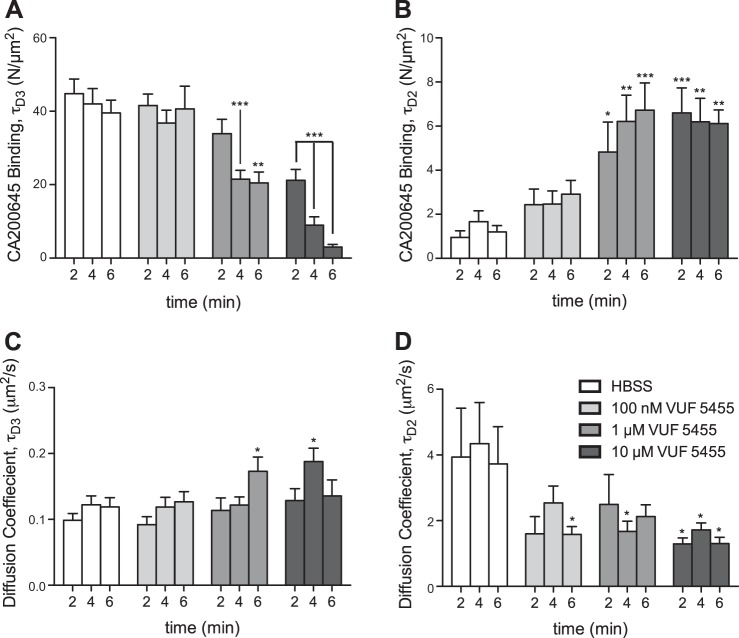
Effect of the allosteric ligand VUF 5455 on the number of CA200645-A_3_AR complexes, by FCS analysis. After a 20 min incubation with 5 nM CA200645 (10 min at 22°C), CHO-A_3_ cells were washed once with HBSS before the medium was replaced with HBSS, with or without the indicated concentrations of the A_3_AR allosteric ligand VUF 5455. FCS measurements were taken on the cell membrane at 2, 4, and 6 min after washout, and subsequent autocorrelation analysis of fluorescence fluctuations allowed quantification of particles with characteristics of τ_D3_ (*A*) and τ_D2_ (*B*), as described in Materials and Methods, along with their diffusion coefficients (*C* and *D*, respectively). Results represent means ± se of 38–40 individual cells taken over 5 independent experiments, analyzed by 1-way ANOVA and the *post hoc* Dunnett multiple-comparison test. *P < 0.05, **P < 0.001, ***P < 0.0001 *vs.* HBSS-only control.

**Figure 6.**
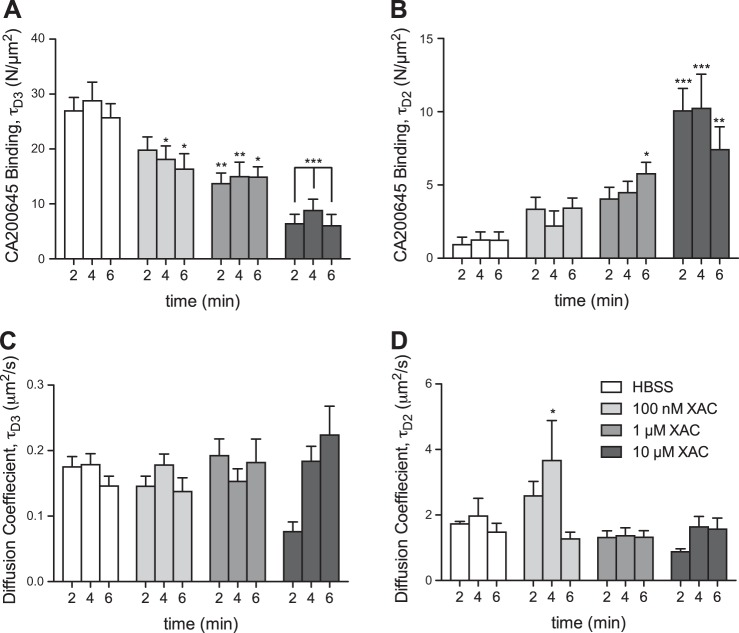
Effect of the orthosteric antagonist XAC on the number of CA200645-A_3_AR complexes, by FCS analysis. After a 20 min incubation with 5 nM CA200645 (10 min, 22°C), CHO-A_3_ cells were washed once with HBSS before the medium was replaced with HBSS, with or without the indicated concentrations of the orthosteric adenosine receptor antagonist XAC. FCS measurements were taken on the cell membrane at 2, 4, and 6 min after washout, and subsequent autocorrelation analysis of fluorescence fluctuations allowed quantification of particles with characteristics of τ_D3_ (*A*) and τ_D2_ (*B*), as described in Materials and Methods, along with their diffusion coefficients (*C* and *D*, respectively). Results represent means ± se of 30–32 individual cells taken over 3 independent experiments and analyzed by 2-way ANOVA and the *post hoc* Dunnett test. *P < 0.05, **P < 0.001, ***P < 0.0001 *vs.* HBSS-only control.

In other studies, we showed, with the use of confocal microscopy, that both allosteric ligands (*e.g.*, VUF 5455; ref. 35) and orthosteric ligands (*e.g.*, XAC) can enhance the dissociation of a fluorescent agonist from whole cells (29, 30). The latter finding provides evidence of negative cooperativity across the interface of A_3_AR homodimers (30). We therefore investigated whether a similar finding could be demonstrated at a subcellular level, by quantifying the 2 slowly diffusing species detected by FCS with a fluorescent antagonist (CA200645). To this end, a series of experiments was performed in which medium was removed from the cells and, after fluorescent ligand was washed out by HBSS exchange, was replaced with HBSS, with or without various concentrations of the A_3_AR allosteric modulator VUF 5455. After the confocal volume was positioned at the upper membrane with an intensity scan, FCS measurements were taken at 2, 4, and 6 min after fluorescent ligand washout. A reduction in the number of slow-diffusing (τ_D3_) antagonist–receptor complexes occurred over time in the presence of VUF 5455, further supporting the idea that this component represents CA200645-A_3_AR complexes (**Fig. 5*A***). This effect was more pronounced at higher concentrations of the allosteric modulator. Interestingly, in parallel with the decrease in the number of τ_D3_ particles, there was a corresponding increase in the number of faster-diffusing τ_D2_ particles (Fig. 5*B*). This finding may indicate that τ_D2_ represents A_3_AR complexes that have released CA200645 during their time in the confocal volume, as such particles appeared to have a shorter apparent dwell time. It is also notable that the τ_D2_ component detected during washout in the absence of added ligand (Figs. 5 and **6**) was lower than when there was no washout (Fig. 3), which suggests that rebinding of CA200645 also contributes to the observation of the τ_D2_ component in the presence of a fluorescent antagonist (see Discussion).

A similar time- and concentration-dependent decrease in particles with τ_D3_ characteristics and a corresponding increase in those with τ_D2_ was observed when the orthosteric adenosine receptor antagonist XAC was used (Fig. 6*A*, *B*), consistent with A_3_ARs existing as dimers on CHO-K1 cell membranes, as we observed earlier (30). Interestingly, there was no major change in the diffusion coefficients for τ_D3_ and τ_D2_ during washout in the presence or absence of VUF 5455 or XAC (Figs. 5 and 6). The increased variability detected in the HBSS control for τ_D2_ is probably accounted for by the low number of particles detected of this component, causing a decrease in signal-to-background ratio and a more variable fit of this particular component (Figs. 5*D* and 6*D*). It is also notable that the pIC_50_ values obtained for XAC from inhibition of the number of τ_D3_ particles and the pEC_50_ for XAC-induced increase in the number of τ_D2_ particles (both ∼6.0) are consistent with the value obtained previously for the IC_50_ for XAC binding to a fluorescent agonist–occupied A_3_AR (30).

### Comparison of the properties of agonist and antagonist receptor complexes

Initial observations showed that only a single diffusing species was detected when diffusion of A_3_AR-GFP was measured and that its diffusion coefficient was equivalent to the τ_D3_ component detected when the CA200645-A_3_AR complexes were measured, in agreement with our findings when using the fluorescent agonist ABEA-X-BY630 (14). These observations are consistent with the hypothesis that τ_D3_ represents diffusion of ligand-A_3_AR complexes, whereas τ_D2_ represents complexes in which there is dissociation of the fluorescent ligand from the A_3_AR during its transit through the confocal volume. However, under equilibrium conditions, where the free ligand is still present, this phenomenon may also reflect ligand association with the receptor during its time in the measurement volume. In the case of the washout experiments with CA200645, the dissociation of the ligand was sufficiently slow (Fig. 5) to allow the contribution of ligand association to τ_D2_ to be largely discounted. These data therefore suggest that τ_D3_ largely reflects the ligand-occupied A_3_AR for both fluorescent agonists and antagonists. If this is the case, then a comparison of the number of particles possessing τ_D3_ characteristics for each fluorescent ligand with the number of particles obtained for A_3_AR-GFP within the same experiment should allow the receptor occupancy to be calculated.

**Figure 7.**
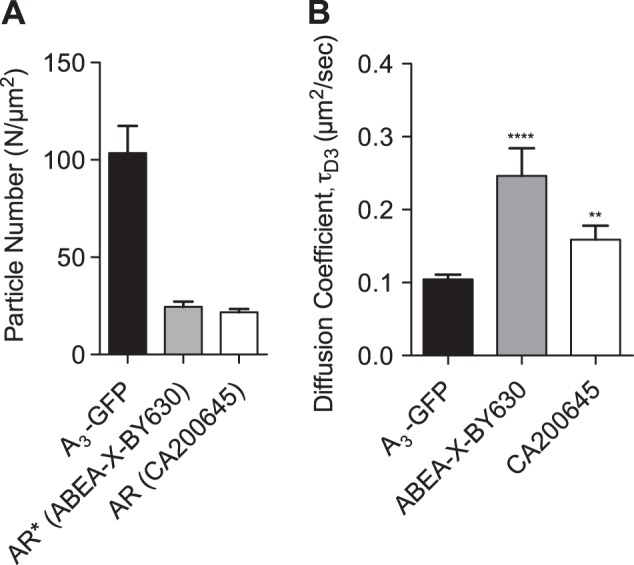
Comparison of the properties of A_3_AR-GFP and agonist– and antagonist–A_3_AR complexes. FCS measurements of the A_3_AR-GFP fusion were taken on the upper membrane of CHO-A_3_AR-GFP cells by using 488 nm excitation (solid bars). Similarly, CHO-A_3_AR-GFP cells were incubated with 5 nM ABEA-X-BY630 (shaded bars) or 5 nM CA200645 (open bars), and FCS analysis of ligand–receptor complexes on the upper cell membrane was used to determine the number and diffusional characteristics of the complexes. Number of particles (for τ_D3_; *A*) and diffusion coefficients (*B*), as determined from FCS measurements of ABEA-X-BY630 and CA200645 τ_D3_ values and the equivalent slowly diffusing GFP component, were compared. Component τ_D2_ was not always detected in the experiments with ABEA-X-BY630 and CA200645, but, in this situation, the diffusional characteristics were compatible with τ_D3_ and are described as τ_D3_ in the figure. Results represent means ± se of 30–32 individual cells taken over 3 independent experiments and analyzed by Student's unpaired *t* test. *P < 0.05, **P < 0.001 *vs.* HBSS-only control.

**Figure 8.**
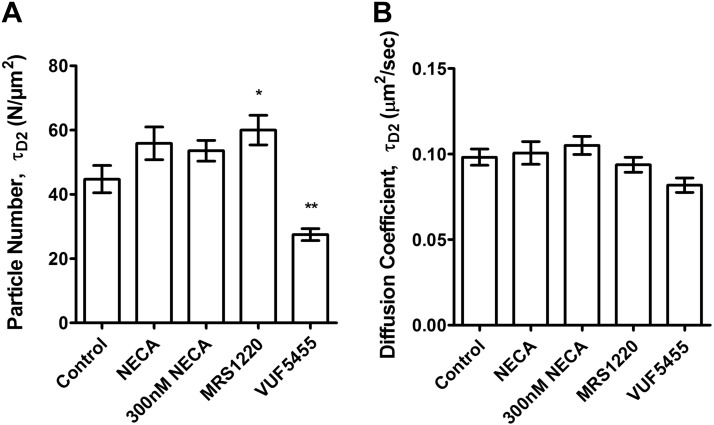
FCS analysis of A_3_AR-GFP after treatment with NECA, MRS 1220, or VUF 5455. Number of particles (*A*) and diffusion coefficient (*B*) were obtained by fitting the autocorrelation curve to a model incorporating two 2-D components, the first representing the photophysical blinking effects of the GFP fluorophore. Data shown for number of particles and diffusion coefficient are for the second (τ_D2_) component that represents the A_3_AR-GFP receptor. Cells were preincubated for 30 min with 300 nM or 10 μM NECA, 1 μM MRS 1220, or 10 μM VUF 5455 at 37°C before FCS measurements. Results represent means ± se of 44–53 individual cells taken over 5 independent experiments and analyzed by 1-way ANOVA with the Dunnett multiple-comparison test. *P < 0.05; **P < 0.01 *vs.* control values.

To determine the relative occupancy levels of CA200645 and the previously described A_3_AR agonist, ABEA-X-BY630, we performed autocorrelation analysis to determine the number of receptor–ligand complexes (τ_D3_ component) for both compounds relative to the total number of A_3_AR-GFP receptors in transfected CHO-K1 cells (103±14/μm^2^; *n*=31). Our analysis revealed that the receptor occupancy levels for the two compounds at the concentration used in the two studies were not significantly different, at 24.5 ± 2.7/μm^2^ (23.7%) and 21.8 ± 1.6/μm^2^ (21.0%) for ABEA-X-BY630 and CA200645, respectively (*n*=31–34; **Fig. 7*A***); however, the diffusion coefficients of ABEA-X-BY630-A_3_AR complexes (0.24±0.02 μm^2^/s) were significantly faster than those observed for the CA200645-A_3_AR complexes in CHO-A_3_AR-GFP cells (0.16±0.04 μm^2^/s) (Fig. 7*B*). The influence of agonists, antagonists, and VUF 5455 on diffusional characteristics of GFP-tagged A_3_ARs was also investigated. However, the diffusion coefficient of A_3_AR-GFP was not significantly affected by treatment with NECA, MRS 1220, or VUF 5455 (**Fig. 8*B***). It was notable, however, that there was a small but significant (*P*<0.05) increase in the number of particles with MRS 1220 and a significant (*P*<0.01) decrease in the number with VUF 5455 (Fig. 8*A*).

## DISCUSSION

The technique of FCS provides a means by which the pharmacological properties of receptor complexes within discrete microdomains of the membrane of a living cell can be interrogated in a quantitative manner (31). FCS analysis of the membrane binding of the fluorescent A_3_AR antagonist CA200645 in CHO-K1 cells expressing the human A_3_AR revealed 2 bound diffusing components, τ_D2_ and τ_D3_, with diffusion coefficients of 2.29 ± 0.35 and 0.09 ± 0.03 μm^2^/s, respectively. These were of similar magnitude, but not identical, to those that had been reported for the fluorescent A_3_AR agonist ABEA-X-BY630 in the same cell line (diffusion coefficients of 2.7 and 0.12 μm^2^/s; ref. 14). It was striking that analysis of the unoccupied GFP-labeled A_3_AR revealed only a single diffusing species with a diffusion coefficient very similar to τ_D3_ detected with CA200645. These data suggest that τ_D3_ represents diffusion of the A_3_AR, although, as previously noted (14, 23), diffusion coefficients of this order are too slow to represent a single receptor molecule and most likely represent a larger oligomeric complex.

Consistent with the assignment of τ_D3_ to A_3_AR–ligand complexes, the binding of CA200645 to this complex could be potently antagonized by nanomolar concentrations of A_3_AR antagonists such as MRS 1220. This effect manifested as a significant reduction in the number of individual fluorescently labeled (τ_D3_) diffusing species detected within the confocal volume after preincubation with unlabeled antagonists (Fig. 4). In marked contrast, the binding of this low concentration of CA200645 (5 nM) was insensitive to inhibition by the agonist NECA at concentrations of up to 300 nM. These data initially appear to be at odds with the data obtained with the fluorescent agonist ABEA-X-BY630, where τ_D3_ was exquisitely sensitive to low nanomolar concentrations of NECA (14). However, this difference most likely represents differential labeling of R and R* states of the receptor by the low concentrations of fluorescent agonist and antagonist used to achieve a low number of particles for FCS experiments (14, 31). It is worth pointing out that the concept of biased agonism suggests that there is a plethora of R* conformations (17).

Selective labeling of high-affinity agonist R* conformations of GPCRs has been a characteristic of studies with radiolabeled agonists (36–41) and is also true of fluorescent ABEA-X-BY630 for the A_3_AR (14). In the same manner, if an antagonist drug has a higher affinity for R compared to R*, then it tends to selectively label R if it is used at low concentrations (36–41). This tendency is particularly the case for compounds that exhibit inverse agonist properties (38–41). In the case of CA200645, the occupancy achieved with the concentrations used in competition FCS experiments was of the order of 21%, with little evidence of a component with higher affinity for agonists. With ABEA-X-BY630 monitored in the same experiments, the occupancy level was similar, but to a conformation that had high affinity for nonfluorescent agonists (ref. 14 and Fig. 7). These data suggest that CA200645 and ABEA-X-BY630 can be used to selectively label the R and R* forms, respectively, by monitoring the τ_D3_-diffusing species of the antagonist- or agonist-bound human A_3_AR. However, one consequence of this interpretation is that the R and R* states cannot readily interconvert within the timescale of these experiments. On the basis of classic ternary complex receptor theory, one would predict that agonist binding to R* would effectively reduce the concentration of R for binding to CA200645 if the 2 conformations are in rapid equilibrium (41). This notion suggests that there is a slow interconversion between the 2 conformations or that there is compartmentalization of the R and R* complexes labeled by CA200645 and ABEA-X-BY630.

We and others have shown that VUF 5455 is an allosteric modulator of the A_3_AR (29, 35, 42). Furthermore, a study with a fluorescent analogue of adenosine in single living cells has shown that VUF 5455 can enhance the dissociation of the labeled ligand from the A_3_AR in a manner consistent with its allosteric mechanism of action (29). To determine whether a similar phenomenon could be demonstrated in discrete membrane microdomains using FCS, we undertook a similar experimental strategy using CA200645 as the labeled ligand. Within the limits of the experimental setup for the FCS studies, CA200645 was shown to dissociate slowly from the A_3_AR (monitored as τ_D3_) with negligible dissociation of ligand occurring within 6 min of removal of CA200645. However, in the presence of increasing concentrations of VUF 5455 during the washout phase of the experiment, there was a marked and concentration-dependent enhancement of the dissociation kinetics of CA200645, consistent with the data obtained in whole cells (29).

**Figure 9.**
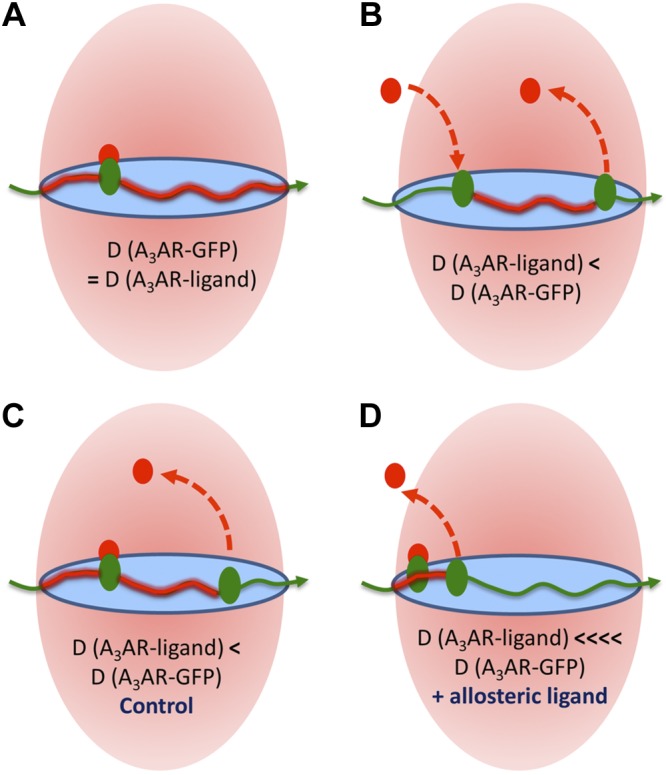
Representation of the ways in which differences in diffusion coefficients (*D* values) may be generated for A_3_AR-GFP and fluorescent ligand-occupied A_3_AR complexes. Pink area represents the confocal volume; blue area at the waist is a representation of the cell membrane within the volume. *A*) If the ligand stays bound to the receptor while it is present in the confocal volume, then the A_3_AR-GFP and A_3_AR–ligand complexes will have the same diffusion coefficients. *B*) If the fluorescent ligand associates with, or dissociates from, the receptor during its time in the measurement volume, then the FCS analysis will conclude that the A_3_AR–ligand complex was present for a shorter time, and a faster diffusion coefficient will be calculated compared to that obtained for A_3_AR-GFP complexes. *C*, *D*) Scenarios for a ligand dissociation experiment where the receptor is prelabeled with fluorescent ligand, and during the washout phase, the ligand dissociates during transit through the volume under control conditions (*C*) or after the addition of an allosteric regulator (*D*). In the experiments shown in Figs. 5 and 6, there is a decrease in the number of particles showing the characteristics of panel *C* and an increase in the number of particles showing the characteristics of panel *D*. Calculation of the diffusion coefficient is necessary to compare the average dwell times of fluorescent species within the confocal volume because of the different sizes of confocal volume illuminated by different laser wavelengths when focused on a diffraction-limited point (such as was used in the FCS experiments). However, for the purposes of this illustration, we assumed that the confocal volumes were the same for the red- and green-labeled species.

It is clear that, in addition to τ_D3_, there is a secondary diffusing species, τ_D2_, that is detected for both fluorescent A_3_AR agonists (14) and antagonists (current study), but not when the unoccupied receptor is monitored (using the A_3_AR-GFP fusion). In the current study, the insensitivity of the τ_D2_ component of CA200645 binding to inhibition by both agonists and antagonists initially suggested that it represents a nonspecific binding component. However, the dissociation experiments undertaken with the allosteric modulator VUF 5455 revealed a significant change in the number of particles detected with a diffusion coefficient compatible with τ_D2_. The simplest explanation of these data is that they represent a diffusional component artificially generated by the dissociation of fluorescent ligand from the receptor species during its transit through the confocal volume (**Fig. 9**). The slow dissociation of CA200645 from the A_3_AR in the absence of the allosteric modulator and the low number of particles with τ_D2_ diffusional characteristics are consistent with this interpretation.

If this hypothesis is correct, then FCS analysis provides a very powerful approach to monitor ligand-binding kinetics in real time and within discrete membrane microdomains of single living cells. Under equilibrium conditions, it is possible that τ_D2_ represents the extent of transient ligand dissociation or, alternatively, association during transit of the receptor through the confocal volume (Fig. 9*B*). The likelihood of the respective contribution of ligand dissociation or association to this fast-diffusing component is dictated by both experimental design (*i.e.*, whether data are collected immediately after addition of fluorescent ligand or after ligand washout) and the binding kinetics of the fluorescent ligand. In competition experiments, however, the sensitivity of τ_D2_ to inhibition by preincubation with nonfluorescent ligands provides some insight into the contribution of fluorescent ligand association to this FCS component. In the case of CA200645, the insensitivity of τ_D2_ to preincubation with nonfluorescent A_3_AR ligands suggests that fluorescent ligand association is not a major contributor to the τ_D2_ component for this ligand. Furthermore, the slow dissociation kinetics of CA200645 (ref. 27 and Fig. 5) make it particularly amenable to the study of unlabeled ligands (*e.g.*, allosteric modulators) that increase ligand dissociation.

To test this concept further, we investigated the effect on τ_D2_ of applying an orthosteric ligand during the washout of CA200645. We showed in earlier work that the A_3_AR exists in homodimeric complexes in CHO cells expressing the human A_3_AR. In addition, we showed in the same study that negatively cooperative interactions exist between the orthosteric binding sites of individual protomers (30). This finding was demonstrated by the ability of orthosteric ligands (adenosine, XAC, and NECA) to enhance the dissociation kinetics of a fluorescent agonist from the A_3_AR (28). Furthermore, this effect was reduced by the increased expression of a ligand-binding–deficient mutant A_3_AR (to increase the proportion of homodimers containing a protomer that is devoid of binding capacity; ref. 30). In the current study, addition of XAC after washout of CA200645 resulted in a marked decrease in the number of particles represented by τ_D3_ and a parallel increase in the number of particles exhibiting the diffusional characteristics of τ_D2_. These results are consistent with negative cooperativity interactions across the A_3_AR dimer interface and provide further evidence that the τ_D2_ component in this experimental paradigm is indicative of A_3_AR complexes that have released the fluorescent ligand during residence in the FCS confocal volume.

These data suggest that the presence and characteristics of the τ_D2_ component of the observed ligand A_3_AR–diffusing species provides important information on the average residence time of fluorescent ligand on the receptor, provided that the observed τ_D2_ is substantially lower than the average time that the receptor spends within the confocal volume (*i.e.*, the FCS dwell time determined using GFP-tagged A_3_ARs). This general concept can be further expanded to explain the small differences in diffusion coefficients for A_3_-GFP, CA200645 (τ_D3_), and ABEA-X-BY630 (τ_D3_) when measured in the same cells (Fig. 7). The simplest explanation of the different diffusion coefficients obtained with fluorescent ligand compared to the mobility of the simultaneously measured GFP-labeled A_3_AR is that they reflect that the life of the ligand–receptor complex is shorter (*i.e.*, dissociation of ligand from the receptor is quicker) than the average dwell time of the A_3_AR in the confocal volume. On this basis, these data indicate that ABEA-X-BY630 dissociates from the A_3_AR far quicker than CA200645, which is consistent with previous observations (14, 27). The data also suggest that the average residence times of ABEA-X-BY630 and CA200645 on the A_3_AR may be reported by τ_D3_ under control conditions when this component has a faster diffusion coefficient (*i.e.*, shorter dwell time in the measurement volume) than that of the A_3_AR-GFP species. Diffusion coefficients must be used to compare the diffusion of red- and green-labeled species in these examples, because the measurement volumes illuminated by 633 nm (red) light is inherently larger than that illuminated by 488 nm (green) laser light. These observations suggest that the actual dwell time (τ_D3_) determined for CA200645 in control conditions represents the average residence time of CA200645 on the receptor, whereas the τ_D2_ levels determined after a 6 min treatment with VUF 5455 or XAC represent allosterically regulated ligand residency times for CA200645. The predicted residence times of these 2 components are presented in **Table 1**. As would be expected in the case of allosteric regulation, the average CA200645 residence time was substantially reduced in the presence of the allosteric regulator VUF 5455 or XAC during fluorescent ligand dissociation (Figs. 5 and 6). It is also important to remember that, because of the low concentrations of CA200645 used in these FCS experiments (5 nM), this allosteric effect is being monitored at the inactive R state of the receptor.

**Table 1.** Predicted average receptor residence times for CA200645 in the absence and presence of ligands that allosterically regulate the A_3_AR

Experiment	HBSS (ms)	+VUF 5455 (ms)	+XAC (ms)
1	249.2 ± 34.9	19.3 ± 14.3	
2	228.1 ± 68.0		18.0 ± 22.1

Data were taken from the apparent dwell times for A_3_AR/CA200645 complexes in the confocal volume measured by FCS analysis in the experiments described in Figs. 5 and 6, from which the diffusion coefficients were calculated (see Materials and Methods). HBSS values were taken from the τ_D3_ component of the measurements made in HBSS medium after a 6 min washout in the presence of HBSS. +VUF 5455 and +XAC data were taken from the τ_D2_ component of the measurements made after a 6 min washout in the presence of 10 μM VUF 5455 (Fig. 5) or 10 μM XAC (Fig. 6). See Figs. 5 and 6 for further experimental details.

The calculation of actual receptor residence times is usually technically difficult when classic binding techniques are used, because of the high potential for rebinding of labeled ligand to the receptor during dissociation from the receptor (from the medium or lipid biophase; refs. 43–45). In the FCS washout experiments performed in this study, the predicted *K*_off_ rate constant could be estimated for a simple ligand–receptor interaction (where there is no interconversion between R and R* within the timescale of the experiment; see above) by taking the reciprocal of the measured dwell time (46). In this case, the dwell time can be taken as the τ_D2_ component during dissociation of the fluorescent ligand (where the A_3_AR is under allosteric regulation and the τ_D2_ values are much shorter than the average dwell time of the A_3_AR in the confocal volume), giving a *K*_off_ of 55.5/s for CA200645, which is much faster than that determined in standard dissociation experiments that measure the residual level of binding at different times in intact cells and membrane fragments (*e.g.*, 0.57/min for fluorescent adenosine at the A_3_AR; ref. 30). One can imagine that although ligand association on the receptor almost certainly occurs, the FCS τ_D2_ dwell time shows the mean residence time on individual receptors. A similar analysis can be undertaken for the τ_D3_ component, yielding a *K*_off_ of 4.02/s, which should represent the off rate from the A_3_AR when there is no allosteric regulation. However, this latter value should be used with caution, since the dwell time (τ_D3_) for CA200645 under normal conditions is close to that predicted for A_3_AR-GFP and may not be sufficiently different to distinguish it with confidence from the time spent by the A_3_AR in the confocal volume. What is clear, however, is that allosteric regulation has dramatically reduced the ligand residence time on the A_3_AR, as we predicted (30).

The suggested interpretation of the nature of τ_D2_ raises the question of why there is a low level of this component present when no nonfluorescent allosteric regulator is present. One interpretation, however, is consistent with this observation. Fig. 3*C* shows that there was clearly an increase in the proportion of τ_D2_ as the concentration of fluorescent CA200645 increased. The demonstration of cooperativity across the A_3_AR dimer interface as an enhanced dissociation of fluorescent ligand by a nonfluorescent orthosteric ligand (this study and ref. 30) relies on the use of low concentrations of the fluorescent probe to occupy only one of the two interacting protomers. When higher concentrations of fluorescent ligand are used, there is an increased probability that both protomers will be occupied by fluorescent ligand, inducing an allosterically enhanced dissociation of fluorescent ligand (which will manifest itself as an increased number of particles demonstrating τ_D2_ diffusion times). The concentration-dependent increase in the number of fluorescent species showing τ_D2_ kinetics is consistent with this hypothesis. In addition, it is clear from Fig. 6 that the decrease in the number of τ_D3_ particles does not exactly match the increase in the number of τ_D2_ particles as the concentration of XAC present during the dissociation phase of the experiment increases. It is likely that, even at the low concentration of fluorescent ligand used in this experiment, a few of the A_3_AR–antagonist complexes will have both protomers occupied by fluorescent ligand (*i.e.*, have double brightness). As a consequence, dissociation of one of these may well have an inordinate influence on the estimated number of τ_D3_ particles that is not exactly balanced by an increase in the number of τ_D2_ particles.

In summary, the current study provides important information on the pharmacological properties of fluorescent antagonist–occupied adenosine A_3_AR complexes. The data obtained indicate that the inactive R conformation of the receptor is selectively monitored by FCS with a fluorescent antagonist ligand and that important insights into ligand binding kinetics, ligand residence time, allosterism, and receptor dimerization can be deduced from careful analysis of the different components of FCS data obtained in discrete membrane domains of single living cells. In particular, the study demonstrated that fluorescent ligand residence times can be monitored by FCS and that these are dramatically reduced by allosterism across the A_3_–AR dimer interface.
